# Multi-Omic Analysis of the Microbiome and Metabolome in Healthy Subjects Reveals Microbiome-Dependent Relationships Between Diet and Metabolites

**DOI:** 10.3389/fgene.2019.00454

**Published:** 2019-05-17

**Authors:** Zheng-Zheng Tang, Guanhua Chen, Qilin Hong, Shi Huang, Holly M. Smith, Rachana D. Shah, Matthew Scholz, Jane F. Ferguson

**Affiliations:** ^1^Department of Biostatistics and Medical Informatics, University of Wisconsin–Madison, Madison, WI, United States; ^2^Wisconsin Institute for Discovery, Madison, WI, United States; ^3^Department of Statistics, University of Wisconsin–Madison, Madison, WI, United States; ^4^Department of Biostatistics, Vanderbilt University Medical Center, Nashville, TN, United States; ^5^Division of Cardiovascular Medicine, Vanderbilt University Medical Center, Nashville, TN, United States; ^6^Division of Pediatric Endocrinology, Children’s Hospital of Philadelphia, Philadelphia, PA, United States; ^7^Vanderbilt Technologies for Advanced Genomics (VANTAGE), Vanderbilt University Medical Center, Nashville, TN, United States; ^8^Vanderbilt Translational and Clinical Cardiovascular Research Center (VTRACC), Vanderbilt University Medical Center, Nashville, TN, United States

**Keywords:** microbiome, diet, metabolome, multi-omics analysis, mediation, interaction

## Abstract

The human microbiome has been associated with health status, and risk of disease development. While the etiology of microbiome-mediated disease remains to be fully elucidated, one mechanism may be through microbial metabolism. Metabolites produced by commensal organisms, including in response to host diet, may affect host metabolic processes, with potentially protective or pathogenic consequences. We conducted multi-omic phenotyping of healthy subjects (*N* = 136), in order to investigate the interaction between diet, the microbiome, and the metabolome in a cross-sectional sample. We analyzed the nutrient composition of self-reported diet (3-day food records and food frequency questionnaires). We profiled the gut and oral microbiome (16S rRNA) from stool and saliva, and applied metabolomic profiling to plasma and stool samples in a subset of individuals (*N* = 75). We analyzed these multi-omic data to investigate the relationship between diet, the microbiome, and the gut and circulating metabolome. On a global level, we observed significant relationships, particularly between long-term diet, the gut microbiome and the metabolome. Intake of plant-derived nutrients as well as consumption of artificial sweeteners were associated with significant differences in circulating metabolites, particularly bile acids, which were dependent on gut enterotype, indicating that microbiome composition mediates the effect of diet on host physiology. Our analysis identifies dietary compounds and phytochemicals that may modulate bacterial abundance within the gut and interact with microbiome composition to alter host metabolism.

## Introduction

The human microbiome is a complex ecosystem of bacteria, viruses, fungi, and bacteriophages, which interact with each other and their host (Sears, 2005; Goodman and Gordon, 2010; Minot et al., 2011). Microbiome composition is unique to an individual, is established early in life, and plays a crucial role in lifelong health (Kau et al., 2011; Minot et al., 2011; Maynard et al., 2012; Koren et al., 2013; Mohammadkhah et al., 2018). Recent discoveries implicating the microbiome in disease have been paradigm-shifting. However, we do not yet understand the molecular mechanisms linking microbiota to health status.

There is considerable site-specificity in microbiome composition, with distinct populations residing within each body site of an individual (Faust et al., 2012; Ding and Schloss, 2014). The relative contributions of the microbiota at each body site to overall host health are not yet clearly defined, but are likely be depend on both the nature of the disease, and the overall health of the host (Zhang et al., 2015). The microbiome composition of the gut is of particular interest, given its location at the crucial interface between exogenous dietary intake and internal nutrient metabolism. Translocation of microbes and microbial metabolites from the intestine to the bloodstream may occur in the absence of intestinal disease, for example during diet-induced post-prandial metabolic endotoxemia (Moreira et al., 2012; Pendyala et al., 2012; Piya et al., 2013). The gut microbiome, in combination with habitual diet, is likely to play a major role in determining gut mucosal membrane permeability and influencing systemic inflammation (Moreira et al., 2012; Pendyala et al., 2012).

Numerous factors determine the specific population of microbiota in humans, with diet being a key contributor (Zeevi et al., 2015; Ferguson et al., 2016). Specific dietary components act as substrates for microbial metabolism, shaping microbiome composition and function. Multiple macronutrient-microbiome associations have been reported, including carbohydrate intake and *Prevotella* abundance (Wu et al., 2011), saturated fat intake and *Bacteroides* and *Faecalibacterium prausnitzii*, and animal protein intake and *Bacteroides* and *Alistipes* (De Filippo et al., 2010; Cotillard et al., 2013; David et al., 2014). Microbiome composition has been linked to disease through modulation of specific metabolites and signaling pathways (Wang et al., 2011; Koeth et al., 2013; Marcobal et al., 2013; Tang et al., 2013). Gut microbial metabolism of animal-product-derived carnitine to the pro-atherogenic metabolite trimethylamine N-Oxide (TMAO) has been found to associate with increased atherosclerotic risk (Wang et al., 2011; Koeth et al., 2013). Many other dietary components may modulate disease risk through parallel mechanisms.

We hypothesized that habitual diet is associated with microbiome composition in healthy humans, and that microbiome composition is associated with gut and plasma metabolites. Using multi-omic sample analysis in up to 150 healthy subjects we profiled the microbiome (16S rRNA; stool and saliva) and the metabolome (stool and plasma) to examine the interaction between diet, the microbiome, and systemic metabolism. Our results identify global relationships and highlight novel associations between specific dietary components and circulating metabolites, that are modulated by gut bacteria, and may have consequences on health status and future disease risk.

## Materials and Methods

### Study Population

The ABO Glycoproteomics in Platelets and Endothelial Cells (ABO) Study recruited healthy volunteers (*N* = 150; men and non-pregnant/lactating women age 18–50) to a protocol at the University of Pennsylvania from 2012–2014. Exclusion criteria included known illnesses, history of organ transplant, tobacco, and prescription medication use (except oral contraceptives). Participants were instructed to avoid over-the-counter medications, supplements, and vitamins for the 2-week period prior to the scheduled visit. Subjects provided a fasting blood sample (following a 12-h overnight fast). As part of a diet and microbiome-focused sub-study, reported here, subjects provided a stool and saliva sample for microbiome analysis (*N* = 136 with stool samples). All subjects completed validated 3-day food records prior to the study visit (Trabulsi and Schoeller, 2001), including on the day directly before the visit, and a weekend day. Nutrient composition was analyzed using Food Processor 8.1 (ESHA Research, Salem, OR). In addition, all subjects completed food frequency questionnaires (FFQ) to assess habitual dietary intake, including serving size, of 134 food items over the previous year [the National Cancer Institute’s Diet History Questionnaire (DHQ I)] (Subar et al., 2001, 2010). Completed subject responses were analyzed using Diet^∗^Calc version 1.5.1. Diet data were converted to nutrient intake values of 191 long-term dietary variables and 139 short-term dietary variables. All subjects provided written informed consent. The study was approved by the Institutional Review Boards of the University of Pennsylvania and Vanderbilt University.

### Sample Processing, DNA Extraction and Sequencing

Subjects collected a stool sample within the 24 h prior to the study visit, using a stool collection kit (Commode Specimen Collection System, Fisher Scientific, Pittsburgh, PA, United States) provided to them. Samples were stored at 4°C and aliquots made within 36 h of sample collection. Processed samples were stored at −80°C prior to nucleic acid extraction. Subjects were instructed to brush their teeth and floss if desired, but not to use mouthwash, following their final meal on the day before the visit (>12 h before visit). Subjects were further instructed not to brush their teeth or use floss or mouthwash on the morning of their visit. Saliva samples were collected using the OMNIGene Discover OM505 DNA/RNA collection kit (DNA Genotek). Following collection, samples were divided into aliquots, and stored at −80°C prior to nucleic acid extraction. DNA was isolated from stool and saliva samples using the PSP Spin Stool DNA Plus Kit (Stratec, Germany). The 16S rRNA gene region was amplified using barcoded primers (Caporaso et al., 2012) (Eurofins Genomics, Louisville, KY, United States) and DNA libraries were cleaned (MinElute PCR Purification kit, Qiagen, Germantown, MD, United States) prior to quantification and pooling. Pooled DNA libraries were sequenced on the MiSeq platform, 300 bp paired-end reads, at an average depth of 158,000 reads/sample (Illumina Inc., San Diego, CA, United States). Stool samples were sequenced in two batches, at the University of Pennsylvania Next-Generation Sequencing Center (UPenn NGSC, *N* = 107) and the Vanderbilt University Technologies for Advanced Genomics (VANTAGE) Core (*N* = 29). All saliva samples (*N* = 85) were sequenced in one batch at VANTAGE. DNA sequences in Fastq files were de-multiplexed, assembled, clustered, and phylogenetically classified using the Mothur pipeline (Schloss et al., 2009). Phylogenetic classification was performed against the Silva V123 16S database. Mothur was run using standard cutoffs, creating OTU clusters at 97% identity.

### Metabolomics

Samples for a subset of individuals (*N* = 75 plasma and *N* = 75 stool, matched subjects) were profiled at Metabolon (Metabolon Inc., Morrisville, NC, United States) using their global metabolomics platform, which can identify and quantitate >1,000 metabolites through multiple mass spectrometry methods. In our study, 812 metabolites were detected in plasma, and 770 in stool samples. For each metabolite, the raw peak intensity was rescaled to set the median across all samples equal to 1, and values below the limit of detection were imputed with the lowest observed value in the dataset. Metabolite pathway enrichment analysis was conducted using MetaboAnalyst (Xia and Wishart, 2011).

### Data Processing for Microbiome, Dietary and Metabolite Variables

Data processing and statistical analysis was performed in R. For the stool microbiome dataset, the OTUs were classified into 11 phyla, 20 classes, 21 orders, 32 families, and 130 genera. For the saliva microbiome dataset, the OTUs were classified into 13 phyla, 21 classes, 32 orders, 52 families, and 103 genera. We obtained two independent measures of dietary intake: 3-day food diaries (for short-term recent diet) and a food frequency questionnaire (FFQ, for long-term habitual diet). Dietary and metabolite variables were normalized using inverse normal transformation (INT) and transformed variables that did not follow a normal distribution (Shapiro–Wilk test *p* < 0.05) were removed (Maritz, 1995). These removed variables had very small variability and/or had many tied observations. The remaining dietary variables were further normalized using the residual method to adjust for total caloric intake and gender, and standardized to have mean of 0 and SD of 1. Since some dietary variables were almost identical, we chose one representative for each highly correlated cluster (Spearman correlation > 0.9), resulting in 91 long-term dietary variables and 82 short-term dietary variables in the final dataset for the downstream analysis. The complete list mapping dietary variables to the selected representative variables are available in Supplementary Tables S1, S2. In order to group metabolites that were highly correlated, we defined metabolic modules using weighted correlation network analysis WGCNA (Langfelder and Horvath, 2008). The WGCNA has been shown to be an efficient and robust method in grouping metabolomic data (McHardy et al., 2013) and allows us to summarize each module by its module eigenvalue. Using WGCNA, the gut metabolites were organized into 8 modules with 40 un-clustered metabolites, and plasma metabolites were organized into 16 modules with 169 un-clustered metabolites. The complete list of metabolites and their module organization are available in Supplementary Tables S3, S4. The abundance values of the un-clustered metabolites were combined with standardized module eigenvalues in the downstream analysis.

### Distance Correlation Analysis

To evaluate the global association between pairs of high-dimensional variables among diet, microbiome and metabolomics, we used the distance correlation *t*-test (Székely and Rizzo, 2013) implemented in the R package “energy” to test the dependence among each pair of these three data types. Compared to Pearson correlation, the distance correlation (Székely et al., 2007; Székely and Rizzo, 2009) is a non-parametric approach (without distributional assumption) and has the power to detect general (non-linear) dependence between two sets of high- dimensional random variables. The distance correlation *t*-test allows the dimension of the random vectors to be larger than the sample size. The ability for detecting general dependence and handling high-dimensionality of data makes distance correlation *t*-test suitable for analyzing this dataset.

### Microbial Enterotypes Analysis

We conducted distance-based clustering using the Partitioning Around Medoids (PAM) method (Kaufman and Rousseeuw, 1987) with the various distances including Euclidean, Bray–Curtis and Jaccard, and identified two enterotypes. To evaluate if diet-metabolite associations are modulated by microbial enterotype, we tested diet-enterotype interaction through linear regression for each pair of diet-metabolite variables, with the metabolite as the outcome, using the individual metabolites rather than metabolite modules.

### Sparse Linear Log-Contrast Model

To further narrow down the interplay between diet/metabolome and microbiome, we used the sparse linear log-contrast model (Lin et al., 2014) to pinpoint important genera that are associated with dietary or metabolite variables. In this model, a dietary or metabolite variable is the response and the top 50 most abundant genera are compositional covariates. For the diet-microbiome analysis, it makes intuitive sense to analyze microbiome variables as the dependent variables since we hypothesize that diet perturbs microbial compositions. Nevertheless, we selected the log-contrast model for several reasons. It is very challenging to find a suitable probabilistic distribution for the microbial composition due to its unique features, such as zero-inflation, over-dispersion, and complex correlation structure (Li, 2015; Tang and Chen, 2018). Further, it has been demonstrated in genetic association studies that such inverse regression (treating dependent variables as covariates) is advantageous if there are multiple dependent variables and the distribution is difficult to specify (Majumdar et al., 2016). Alternative methods that treat microbiome as dependent variables include sparse Dirichlet-Multinomial (DM) method (Chen and Li, 2013) and multivariate zero-inflated logistic-normal method (Li et al., 2018), however, we determined that the log-contrast model was the most suitable currently available model for our study. For the taxa that are unclassified at the genus level, their identities at higher levels were used. Because of the unit-sum constraint of the microbial relative abundance, the components of a composition cannot vary freely. The sparse linear log-contrast model respects the compositional nature of the microbiome data, in which the unit-sum constraint on the compositional vector is translated into the zero-sum constraint on the association coefficients across taxa in log-ratio scale (Lin et al., 2014). The zero-sum constraint is crucial for the resulting estimator to enjoy interpretive advantages over a standard lasso estimator (Tibshirani, 1994). In our analysis, we used 10-fold cross validation to choose the tuning parameter. To obtain stable selection results, we generated 100 bootstrap samples and used the same cross-validation procedure to select the genera. The genera that were selected over 70 times out of 100 were considered associated with the dietary or metabolite variable.

### Microbiome Mediation Analysis

We considered how the effect of a dietary nutrient on a metabolite is transmitted through the microbial communities. Specifically, we were interested in identifying microbial taxa that mediate the diet-metabolite pathway. We focused on pairs of diet-metabolite variables linked to at least one common genus identified by the log-contrast model in section 2.7, and applied mediation analysis to the diet-gut microbiome-metabolite triplet. The top 50 most abundant genera were used as candidate microbiome mediators. To handle the compositional and high-dimensional nature of microbiome mediators, we utilized the state-of-the-art compositional mediation analysis for microbiome data (R Package ccmm) (Sohn and Li, 2019). Certain assumptions are required to make casual interpretation of the mediation effects (Imai et al., 2010; Sohn and Li, 2019). In particular, the key assumption assumes that there is no unmeasured confounding variable after controlling covariates. The method enables us to estimate the total mediation effects of microbiome composition, as well as to select important microbial taxa mediating the diet-metabolite association and estimate taxon-specific mediation effects.

**FIGURE 1:**
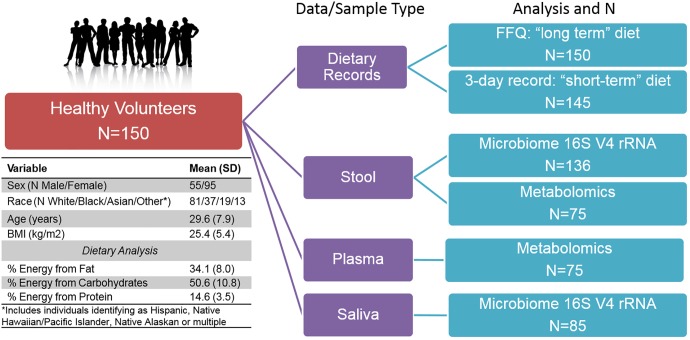
Overview of Study design, subject characteristics, and multi-omic sample availability.

## Results

We conducted multi-omic phenotyping of up to 150 healthy subjects to probe diet, microbiome, and metabolome relationships in a cross-sectional sample. The overall study design, sample availability and subject characteristics are shown in Figure 1. By design, participants were healthy with no overt disease, consuming diets broadly representative of a standard American diet. Dietary variables calculated from the short and long-term diet questionnaires were significantly correlated with each other, suggesting that subjects’ diets immediately prior to microbiome sampling were broadly representative of their diets over the past year. Of 150 enrolled subjects who completed a dietary questionnaire, 136 subjects provided a stool sample for microbiome analysis. We conducted metabolomic profiling in matched stool and plasma samples in a subset of these individuals (*N* = 75) and collected saliva samples for microbiome analysis in a separate subset (*N* = 85). No global associations were detected between diet, the microbiome, or metabolome, and demographic variables (age, sex, race, and BMI; PERMANOVA *p* > 0.1). We observed a difference in gut microbiome composition by batch (*p* = 0.04, UPenn vs. VANTAGE, see section “Sample Processing, DNA Extraction and Sequencing”). There were no differences in metabolite or nutrient profiles between the batches (*p* > 0.1), or in enterotype distribution (chi-square test *p* = 0.86). To assess whether the batch effect had any effect on our results, we repeated all the relevant analyses using only batch 1 samples (*N* = 107) and confirmed the conclusions remained the same. As the overall results did not differ, we report here the results from the analyses of the entire sample.

### The Gut Microbiome Is Related to Diet and Metabolites on a Global Level

**FIGURE 2:**
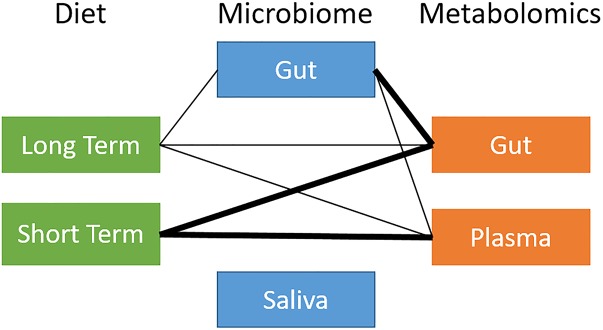
Overview of global relationships between microbiota, diet, and metabolites. Thick line: distance correlation *t*-test *p*-value *<* 10^−5^; thin line: distance correlation *t*-test 10^−5^ *< p*-value < 10^−1^.

We ran a global analysis using distance correlation *t*-test to obtain an integrated view of the relationships and relative importance of dietary measures (short-term and long-term diet), microbiome body site samples (stool and saliva), and metabolites (stool and plasma). As shown in Figure 2, there were considerable inter-relationships, with particularly strong associations between the gut microbiome and the gut metabolome (*p* = 2.2 × 10^−10^), and between long-term diet and the gut microbiome (*p* = 7.8 × 10^−4^). Short-term diet was significantly associated with the gut and plasma metabolome (*p* < 1 × 10^−3^), but not the microbiome. We found no global associations between the saliva-derived oral microbiome and other data types. Within data types, there was very strong global correlation between short- and long-term diet (*p* < 1 × 10^−15^), and between stool and plasma metabolites (*p* = 2.1 × 10^−8^), but not between the gut and oral microbiome (*p* = 0.7). Based on the evidence in the global analysis, we decided to focus our remaining analyses on the gut microbiome and long-term diet, and to evaluate their interplay with gut and circulating metabolites.

### Dietary Nutrients Are Associated With Gut Microbes

**FIGURE 3:**
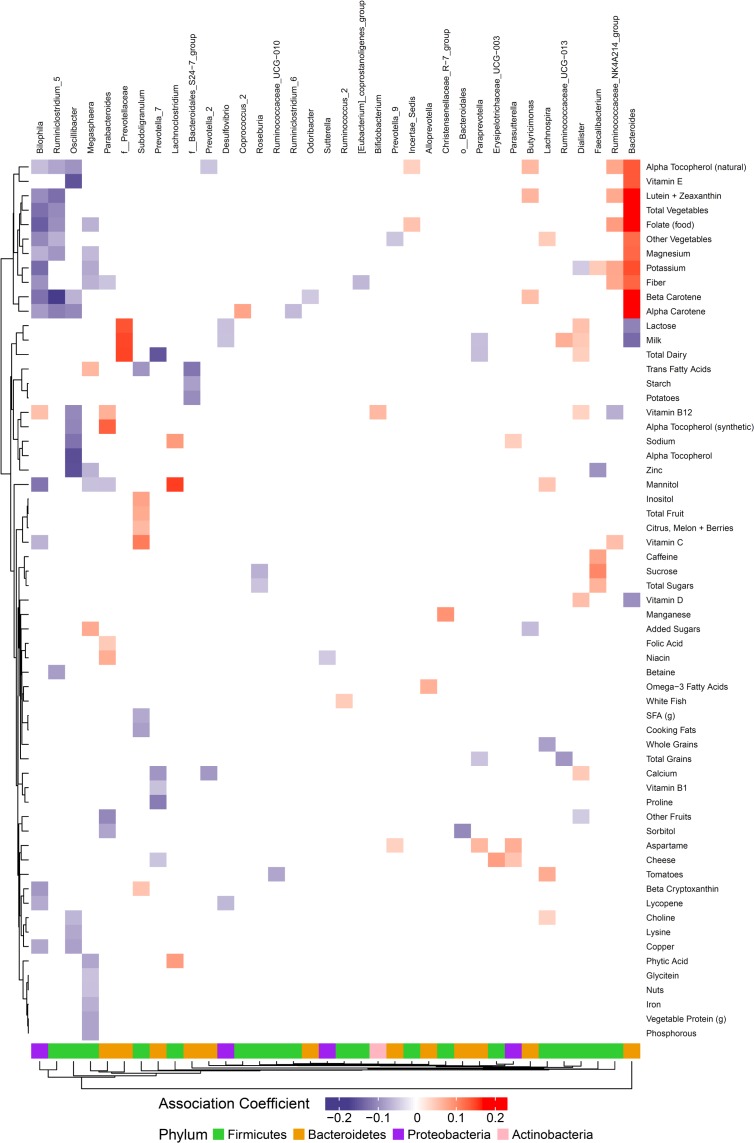
Associations between habitual dietary nutrient intake and gut microbiome. Color intensity reflects the magnitude of the association coefficients between dietary variables and taxa.

We hypothesized that gut microbiome composition would vary based on the intake of specific nutrients. From the sparse log-contrast model, we identified 61 (67%) long-term dietary nutrients associated with at least one bacterial genus (Figure 3). Several nutrients associated with three or more genera, as shown in Table 1. These dietary nutrients were predominately found in plant-derived foods and dairy products, suggesting that inclusion or exclusion of these food groups in the diet may be particularly important in the modulation of gut microbiome composition.

### Circulating and Gut Metabolites Are Associated With Gut Microbes

**FIGURE 4:**
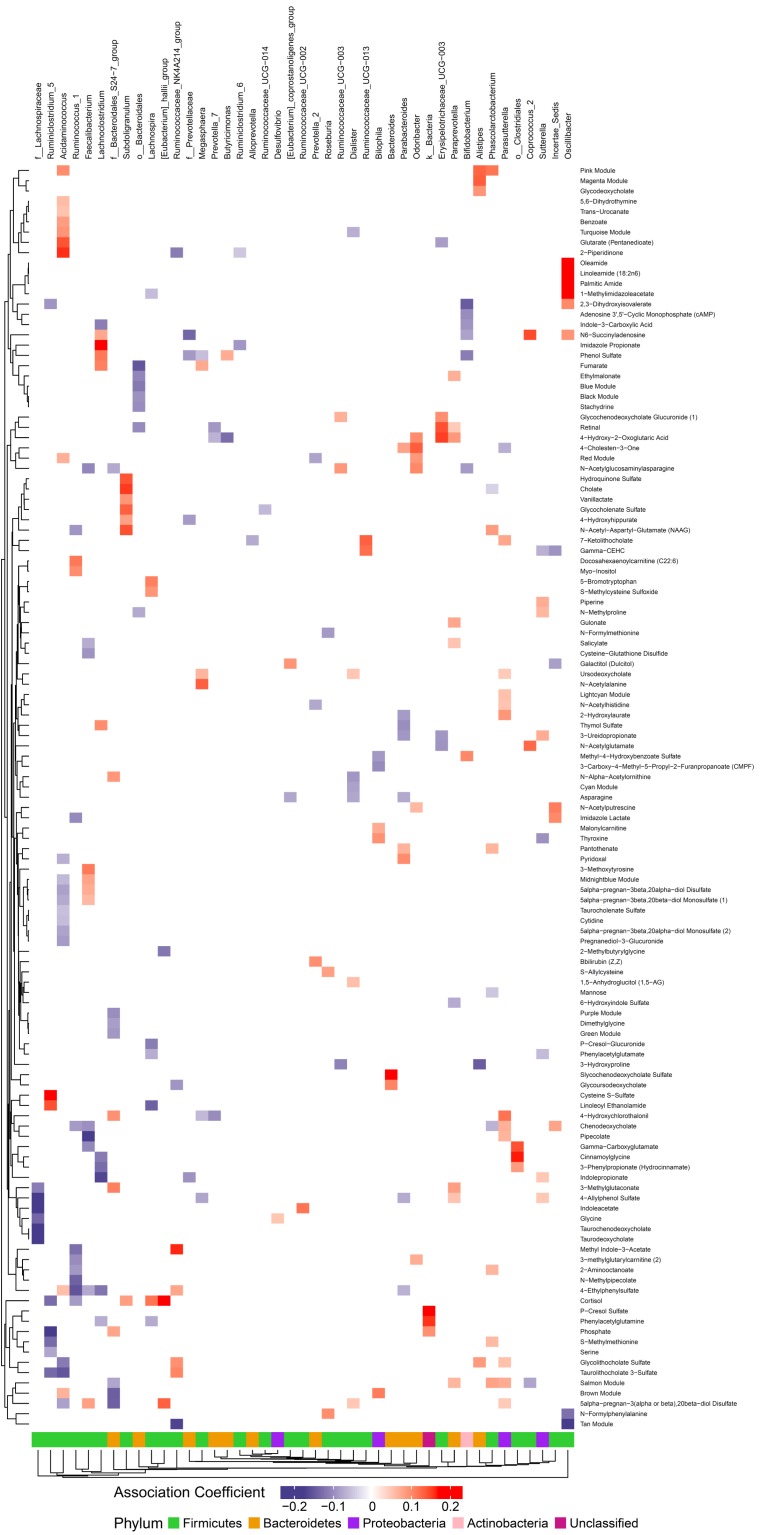
Associations between gut microbiome and metabolites in plasma. Color intensity reflects the magnitude of the association coefficients between metabolites and taxa.

**FIGURE 5:**
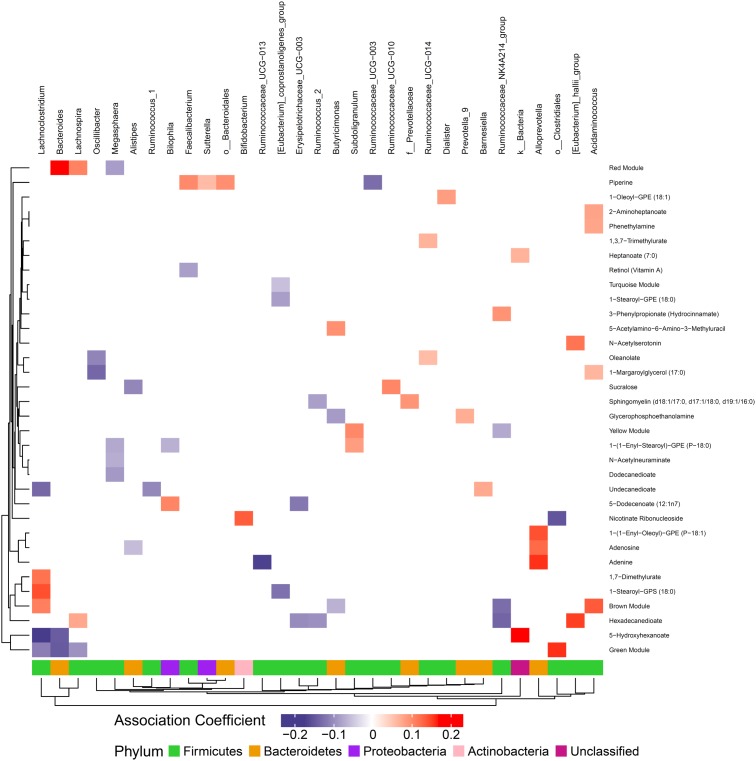
Associations between gut microbiome and metabolites in stool. Color intensity reflects the magnitude of the association coefficients between metabolites and taxa.

We hypothesized that gut microbiome composition would associate with specific metabolites in the gut and circulation, reflecting taxon-specific metabolism. We identified 123 (66%) circulating metabolite variables and modules and 34 (71%) gut metabolite variables and modules that associated with at least one bacterial genus (Figure 4, 5). Several metabolites were associated with multiple genera, as shown in Table 2. Of these highly bacterial-related metabolites, many have known functions in bile acid metabolism, lipid and amino acid metabolism, or metabolism of xenobiotics, highlighting the important role of microbes in modulating host metabolism in key pathways.

### Gut Bacterial Taxa Mediate the Association Between Dietary Nutrients and Metabolites

We were interested in whether gut bacterial taxa mediate the relationship between diet and metabolites. Mediation analysis revealed multiple taxa influencing the association between dietary intake and metabolites in plasma or stool. Given the inter-relationships between metabolic variables, we were interested in which pathways were most affected by microbiome mediation. We identified metabolic pathways with evidence for strong diet-microbiome effects, defined as having 3 or more metabolites in a sub-pathway with significant diet associations mediated by the microbiome, or association with a metabolite module (Table 3). These included amino acid metabolism (histidine, phenylalanine, and tyrosine), lipid metabolism (fatty acids, bile acids, and steroids), and xenobiotics (benzoate, and food components). Of the dietary variables, plant-derived nutrients (vitamins and phytochemicals) and metals were strongly represented. Our data suggest that metabolic flux through these pathways is particularly susceptible to interaction between dietary intake and microbiome composition.

**Table 1:** Long term intake of dietary nutrients associated with at least three gut microbial taxa.

Dietary nutrient	Primary food source	Bacterial taxon^∗^	
		Positive association	Negative association
Alpha Carotene	Plants	*Bacteroides, Coprococcus 2*	*Bilophila, Ruminiclostridium 5, Ruminiclostridium 6, Oscillibacter*
Beta Carotene		*Bacteroides, Butyricimonas*	*Bilophila, Odoribacter, Ruminiclostridium 5, Oscillibacter*
Lutein and Zeaxanthin		*Bacteroides, Ruminococcaceae NK4A214, Butyricimonas*	*Bilophila, Ruminiclostridium 5*
Vegetables		*Bacteroides, Lachnospira*	*Prevotella 9, Bilophila, Ruminiclostridium 5*
Vitamin E		*Bacteroides, IncertaeSedis, Ruminococcaceae NK4A214, Butyricimonas*	*Bilophila, Prevotella 2, Ruminiclostridium 5, Oscillibacter*
Vitamin C		*Subdoligranulum, Ruminococcaceae NK4A214*	*Bilophila*
Vitamin B12		*Parabacteroides, Bilophila, Dialister, Bifidobacterium*	*Ruminococcaceae NK4A214, Oscillibacter*
Folate		*Bacteroides, Incertae Sedis, Ruminococcaceae NK4A214*	*Bilophila, Ruminiclostridium 5, Megasphaera*
Dietary Fiber		*Bacteroides, Ruminococcaceae NK4A214*	*Parabacteroides, [Eubacterium]coprostanoligenes, Bilophila, Megasphaera*
Milk	Dairy products	*Dialister, Ruminococcaceae UCG-013*, f Prevotellaceae	*Bacteroides, Paraprevotella, Desulfovibrio*
Cheese		*Parasutterella, Erysipelotrichaceae UCG-003*	*Prevotella 7*
Calcium	Dietary Metals	*Dialister*	*Prevotella 7, Prevotella 2*
Zinc			*Faecalibacterium, Megasphaera, Oscillibacter*
Sodium		*Parasutterella, Lachnoclostridium*	*Oscillibacter*
Magnesium		*Bacteroides*	*Bilophila, Ruminiclostridium 5, Megasphaera*
Potassium		*Bacteroides, Faecalibacterium, Ruminococcaceae NK4A214*	*Bilophila, Dialister, Megasphaera*
Aspartame	Processed foods	*Prevotella 9, Parasutterella, Paraprevotella*	
Mannitol		*Lachnospira, Lachnoclostridium*	*Parabacteroides, Bilophila, Megasphaera*
Trans Fat		*Megasphaera*	*Subdoligranulum, f Bacteroidales S24-7*

*^∗^Bacterium reported as genus, unless otherwise specified (f, family; c, class; o, order; p, phylum; k, kingdom).*

### Differences in Abundance of Metabolites by Gut Microbial Enterotype

We identified two gut microbiome enterotypes in our sample, with good separation of the sub-groups by Principal Coordinates Analysis (PCoA) using the Jaccard distance (see Supplementary Figure S1). There were 54 individuals categorized as Enterotype 1, and 82 individuals categorized as Enterotype 2. There was no difference in age or race distribution across enterotypes, or in sequencing batch, although there was a trend toward a higher proportion of women in enterotype 2 (52% vs. 69% female, chi-square test *p* = 0.054). Individuals in enterotype 2 had lower BMI (26.9 vs. 24.5, *p* = 0.01). The primary differentiating characteristic between the two gut enterotypes was in the abundance of family Ruminococcaceae, with significantly higher proportion of Ruminococcaceae in enterotype 2 (Supplementary Figure S2). Analysis of metabolites by enterotype revealed striking differences between the groups: 112 plasma metabolites and 122 stool metabolites were significantly different by enterotype (unadjusted *p* < 0.05, Supplementary Tables S5, S6). Unadjusted *p*-values are reported in the enterotype analysis because the analysis used individual metabolites rather than metabolite modules and many metabolites are highly correlated. While the enterotype-associated metabolites spanned many biological pathways, they were enriched in certain categories. We selected all nominally associated metabolites for pathway enrichment analysis. Plasma metabolites that differed by enterotype were significantly enriched for amino acid metabolism (*p* < 0.05), particularly the essential amino acids phenylalanine, tryptophan, and tyrosine, the essential branched-chain amino acids valine, leucine and isoleucine, as well as arginine and proline. Stool metabolites differing by enterotype were enriched in taurine and niacin (vitamin B3) metabolism (*p* < 0.05). Individuals in Enterotype 1 had slightly higher alcohol and cholesterol consumption than Enterotype 2 (*p* < 0.05), but there were otherwise limited differences in dietary intake by enterotype, suggesting that the metabolite differences were not solely attributable to differences in diet.

### Gut Microbial Enterotype Modulates the Relationship Between Diet and Metabolites

As observed in the mediation analysis for individual taxa, microbiome composition mediates the association between dietary nutrients and metabolites. We hypothesized that gut enterotype, as a composite measure of microbiome differences, would modify the relationship between dietary nutrient intake and downstream metabolism. We found evidence for significant interaction between habitual dietary intake and gut enterotype on plasma and stool metabolites across many classes of nutrients and metabolites. Of diet-metabolite pairs that were enterotype-dependent, the most frequent dietary components, which associated with >100 metabolites each, included plant-derived nutrients (fiber, carotenoids, and isoflavones) and artificial sweeteners (saccharin, mannitol, aspartame, and xylitol), as well as animal protein, trans fatty acids, caffeine, and alcohol. The diet- and enterotype-dependent metabolites spanned many pathways, but the metabolites with the most frequent associations with dietary variables (>30 dietary variables each) were predominately bile acids and xenobiotic metabolites in plasma, and xenobiotic and amino acid metabolites in stool.

**FIGURE 6:**
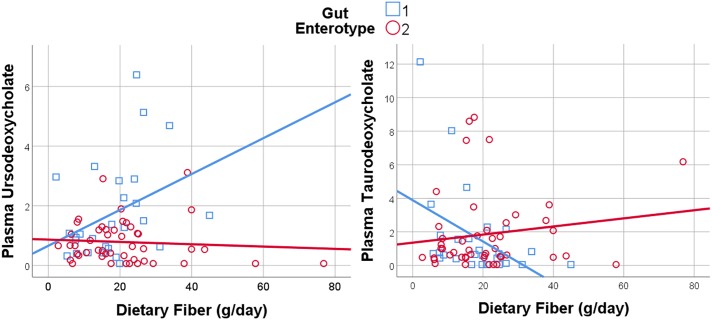
Dietary Fiber has a gut enterotype-dependent association with plasma secondary bile acids including ursodeoxycholate and taurodeoxycholate.

**FIGURE 7:**
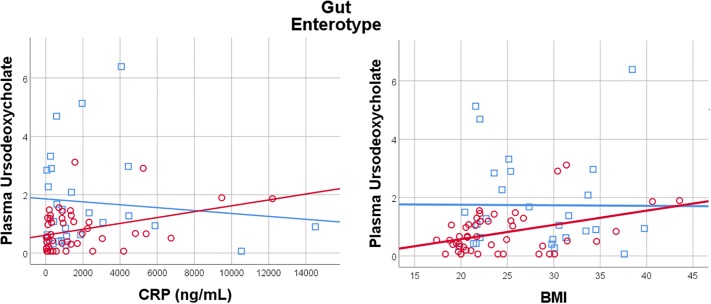
Plasma Ursodeoxycholate has a gut enterotype-dependent relationship with plasma C-Reactive Protein and BMI, with a positive association in Enterotype 2, and no relationship in Enterotype 1.

Given the importance of bile acids in both gut metabolism and cardiometabolic disease risk, we were particularly interested in the observed microbiome-mediated effects of diet on bile acid signaling. As shown in Figure 6, habitual intake of dietary fiber was associated with higher plasma ursodeoxycholate in individuals with enterotype 1, but there was no relationship between diet and ursodeoxycholate in enterotype 2. Conversely, high dietary fiber was associated with decreased plasma taurodeoxycholate in individuals with enterotype 1, and slightly increased levels in enterotype 2. Many of the circulating bile acids were highly correlated with each other, and as such the results for taurodeoxycholate represent similar significant associations for dietary fiber with taurocholate, taurolithocholate 3 sulfate, glycolithocholate, glycolithocholate sulfate, taurochenodeoxychlate, glycodeoxycholate, glycocholate, and glycodeoxycholate sulfate, (Spearman correlation > 0.5 for metabolite pair, and *p* < 0.05 for enterotype-mediated association with diet). Of note, dietary choline was highly correlated with dietary fiber (Spearman correlation 0.7), reflecting some overlapping food sources and dietary patterns, and similar patterns of association with bile acids were also observed for choline. Interestingly, there was a modest positive relationship between plasma ursodeoxycholate (*p* < 0.05), but not plasma taurodeoxycholate, and plasma C-Reactive Protein (CRP) and BMI in individuals with enterotype 2, but not in enterotype 1 (Figure 7). These data suggest that individuals with enterotype 1 have bile acid metabolism that is highly diet-responsive, whereas individuals with enterotype 2 have bile acid production which is less sensitive to differences in dietary intake, but may be more likely to relate to poor metabolic health.

**Table 2:** Plasma and stool metabolites associated with three or more gut microbial taxa.

Metabolite	Metabolic function	Bacterial Taxon^∗^	
		Positive association	Negative association
**Plasma metabolites**			
Chenodeoxycholate	Primary bile acid	*Parasutterella, Incertae Sedis*	*Phascolarctobacterium, Faecalibacterium, Ruminococcus 1*
Glycolithocholate sulfate	Secondary bile acid	*Alistipes, Parasutterella, Ruminococcaceae NK4A214*	*Acidaminococcus*
7-ketolithocholate		*Parasutterella, Ruminococcaceae UCG-013*	*Alloprevotella*
Taurolithocholate 3-sulfate		*Ruminococcaceae NK4A214*	*Acidaminococcus, Ruminiclostridium 5*
Ursodeoxycholate		*Parasutterella, Dialister, Megasphaera*	
4-cholesten-3-one	Lipid	*Parabacteroides, Odoribacter*	*Parasutterella*
5alpha-pregnan-3(alpha or beta), 20beta-diol disulfate		*Parasutterella, Dialister, Faecalibacterium, [Eubacterium]hallii*	*Acidaminococcus, f Bacteroidales S24-7*
Cortisone		*Subdoligranulum, Lachnospira, [Eubacterium]hallii*	*Ruminococcus 1, Ruminiclostridium 5*
4-hydroxy-2-oxoglutaric acid		*Paraprevotella, Odoribacter, Erysipelotrichaceae UCG-003*	*Prevotella 7, Butyricimonas*
Phenol sulfate	Amino acid	*Butyricimonas, Lachnoclostridium*	*Bifidobacterium, Megasphaera, f Prevotellaceae*
Asparagine			*Parabacteroides, [Eubacterium]coprostanoligenes, Dialister*
*N*-acetyl-aspartyl-glutamate (NAAG)		*Phascolarctobacterium, Subdoligranulum*	*Ruminococcus 1*
3-methylglutaconate		*Paraprevotella, f Bacteroidales S24-7*	*f Lachnospiraceae*
Indolepropionate		*Sutterella*	*f Prevotellaceae, Lachnoclostridium*
Phenylacetylglutamine		*k Bacteria*	*Lachnospira, Lachnoclostridium*
*N*-acetylglucosaminylasparagine		*Odoribacter, Ruminococcaceae UCG-003*	*Bifidobacterium, Faecalibacterium, f Bacteroidales S24-7*
Phosphate	Energy	*k Bacteria, f Bacteroidales S24-7*	*Ruminiclostridium 5*
Fumarate		*Megasphaera, Lachnoclostridium*	*o Bacteroidales*
N6-succinyladenosine	Nucleotide	*Coprococcus 2, Oscillibacter, Lachnoclostridium*	*Bifidobacterium, f Prevotellaceae*
3-ureidopropionate		*Sutterella*	*Parabacteroides, Erysipelotrichaceae UCG-003*
4-ethylphenylsulfate	Xenobiotic	*Acidaminococcus, Ruminococcaceae NK4A214*	*Parabacteroides, Faecalibacterium, Ruminococcus 1, Lachnoclostridium*
4-allylphenol sulfate		*Paraprevotella, Sutterella*	*f Lachnospiraceae, Parabacteroides, Megasphaera*
4-hydroxychlorothalonil		*Parasutterella, f Bacteroidales S24-7*	*Prevotella 7, Megasphaera*
Retinal		*Paraprevotella, Erysipelotrichaceae UCG-003*	*o Bacteroidales, Prevotella 7*
2,3-dihydroxyisovalerate		*Oscillibacter*	*Bifidobacterium, Ruminiclostridium 5*
2-piperidinone		*Acidaminococcus*	*Ruminiclostridium 6, Ruminococcaceae NK4A214*
Gamma-CEHC		*Ruminococcaceae UCG-013*	*Incertae Sedis, Sutterella*
Salmon module		*Parasutterella, Phascolarctobacterium, Paraprevotella*	*f Bacteroidales S24-7, Coprococcus 2*
Brown module		*Bilophila, Acidaminococcus*	*f Bacteroidales S24-7*
Pink module		*Alistipes, Phascolarctobacterium, Acidaminococcus*	
Red module		*Odoribacter, Acidaminococcus*	*Prevotella 2*
**Stool metabolites**			
Hexadecanedioate	Lipid	*Lachnospira, [Eubacterium]hallii*	*Ruminococcus 2, Ruminococcaceae NK4A214, Erysipelotrichaceae UCG-003*
Undecanedioate		*Barnesiella*	*Ruminococcus 1, Lachnoclostridium*
3-hydroxyhexanoate		*k Bacteria*	*Bacteroides, Lachnoclostridium*
1-(1-enyl-stearoyl)-GPE (P-18:0)		*Subdoligranulum*	*Bilophila, Megasphaera*
Piperine	Xenobiotic	*o Bacteroidales, Sutterella, Faecalibacterium*	*Ruminococcaceae UCG-003*
Brown module		*Acidaminococcus, Lachnoclostridium*	*Ruminococcaceae NK4A214, Butyricimonas*
Green module		*o Clostridiales*	*Bacteroides, Lachnospira, Lachnoclostridium*
Red module		*Bacteroides, Lachnospira*	*Megasphaera*

*^∗^Bacterium reported as genus, unless otherwise specified (f, family; c, class; o, order; p, phylum; k, kingdom).*

**Table 3:** Diet and microbiome mediated metabolites.

Dietary nutrient	Metabolite	Tissue	Pathway	Sub-pathway	Bacterial taxon^∗^
Copper, lysine, vitamin E	1-methylimidazoleacetate	Plasma	Amino acid	Histidine metabolism	*Oscillibacter*
Sodium, phytic acid	Imidazole propionate	Plasma			*Ruminiclostridium_6, Oscillibacter, Lachnoclostridium*
Cheese, sodium, vitamin E	*N*-acetylhistidine	Plasma			*Parasutterella, Prevotella_2*
Sugar	3-methoxytyrosine	Plasma		Phenylalanine and tyrosine metabolism	*f__Ruminococcaceae*
Vegetables, tomato	5-bromotryptophan	Plasma			*Lachnospira*
Sugar	*N*-formylphenylalanine	Plasma			*Roseburia*
Lutein + zeaxanthin	Phenol sulfate	Plasma			*Butyricimonas*
Vegetables	Thyroxine	Plasma			*Parasutterella*
Vitamin E, B carotene, folate, lutein + zeaxanthin, copper	3-carboxy-4-methyl-5-Propyl-2-furanpropanoate	Plasma	Lipid	Fatty acid, dicarboxylate	*Butyricimonas, Oscillibacter*
Vitamin E, B carotene, lutein + zeaxanthin, grains, proline, vitamin B1, dairy, calcium, cheese	4-hydroxy-2-oxoglutaric acid	Plasma			*Butyricimonas, Erysipelotrichaceae_UCG-003, Paraprevotella, Prevotella_7*
Sugar, mannitol, fiber, folate, glycitein, iron, magnesium, potassium, phosphorous, phytic acids, nuts,	Dodecanedioate	Stool			*Megasphaera*
Vitamin E, folate, lutein + zeaxanthin, cheese, tomatoes	Hexadecanedioate	Stool			*Bifidobacterium, Butyricimonas, Ruminococcaceae_UCG-002, Erysipelotrichaceae_UCG-003*
Sodium	Undecanedioate	Stool			*Prevotella_2*
Sugar, sodium, cheese, zinc	Chenodeoxycholate	Plasma		Primary bile acid metabolism	*Parasutterella, Phascolarctobacterium, Incertae_Sedis, Faecalibacterium*
SFA (g)	Cholate	Plasma			*Phascolarctobacterium*
Cheese	Glycochenodeoxycholate Glucuronide	Plasma			*Erysipelotrichaceae_UCG-003*
Vegetables	Glycochenodeoxycholate Sulfate	Plasma			*Bacteroides*
Cheese, sodium	7-Ketolithocholate	Plasma		Secondary bile acid metabolism	*Parasutterella*
Cooking fats	Glycocholenate sulfate	Plasma			*Butyricimonas*
Potassium, folate, lutein +zeaxanthin, vitamin B12, cheese, sodium	Glycolithocholate Sulfate	Plasma			*Acidaminococcus, f__Prevotellaceae, Parasutterella, Ruminococcaceae_NK4A214_group*
B carotene, folate, vegetables	Glycoursodeoxycholate	Plasma			*Megasphaera*
Potassium, folate, lutein + zeaxanthin, vitamin B12, A carotene, B carotene	Taurolithocholate 3-sulfate	Plasma			*Acidaminococcus, Ruminiclostridium_5, Ruminococcaceae_NK4A214_group*
Sugar, fiber, folate, glycitein, magnesium, mannitol, nuts, potassium, vegetables, cheese, calcium, sodium	Ursodeoxycholate	Plasma			*Megasphaera, Parasutterella, k__Bacteria*
Zinc, calcium, lactose, dairy, cheese, sodium, sugar, fruit, potatoes, starch, trans fat, vitamin B12, vitamin D	5α-pregnan-3(α or β),20β-diol disulfate	Plasma		Steroid/sterol	*[Eubacterium]_coprostanoligenes_group, f__Bacteroidales_S24-7_group, Erysipelotrichaceae_UCG-003*
Zinc	5α-pregnan-3β,20β-diol monosulfate	Plasma			*[Eubacterium]_coprostanoligenes_group*
Cooking fats	Cortisol	Plasma			*Butyricimonas*
Fruit, cheese, sodium, niacin	4-cholesten-3-One	Plasma			*Parabacteroides*
Phytic acid, niacin, fruit, sugar	4-ethylphenylsulfate	Plasma	Xenobiotics	Benzoate metabolism	*Lachnoclostridium, Parabacteroides, Ruminococcus_1*
Total Dairy	4-hydroxyhippurate	Plasma			*Paraprevotella*
Lutein + zeaxanthin, folate, vitamin E, B carotene, copper	Methyl-4-hydroxybenzoate sulfate	Plasma			*Bilophila, Bifidobacterium*
B carotene, folate, lutein + zeaxanthin, sodium, copper, lysine, vitamin E	2,3-dihydroxyisovalerate	Plasma		Food component/plant	*Bifidobacterium*
Folate, potassium, vitamin B12	2-piperidinone	Plasma			*Acidaminococcus, Ruminococcaceae_NK4A214_group*
Sugar, sorbitol, mannitol, glycein, iron, potassium, phosphorous, nuts, fiber, folate, magnesium, vegetables, niacin, fruit, dairy	4-allylphenol sulfate	Plasma			*Megasphaera, f__Prevotellaceae, Ruminiclostridium_6, Paraprevotella, Parabacteroides*
Lutein + zeaxanthin, vitamin B12, vitamin E, folate	Methyl indole-3-acetate	Plasma			*Ruminococcaceae_NK4A214_group, Butyricimonas*
Zinc, sucrose	Piperine	Stool			*Faecalibacterium, Parasutterella*
Proline, grains, vitamin B1, dairy, cheese, sorbitol	Retinal	Plasma			*Erysipelotrichaceae_UCG-003, Paraprevotella, o__Bacteroidales*
Sorbitol	Stachydrine	Plasma			*o__Bacteroidales*
Niacin, fruit	Thymol sulfate	Plasma			*Parabacteroides*
Sorbitol	Black, blue module	Plasma	Module		*o__Bacteroidales*
Copper, folate, lutein + zeaxanthin, B carotene, vitamin E, potatoes, starch, trans fat, vitamin B12	Brown module	Plasma			*Bilophila, Butyricimonas, f__Bacteroidales_S24-7_group*
Sugar, mannitol, fiber, phytic acid, potassium, vitamin B12, B carotene, folate	Brown module	Stool			*[Eubacterium]_coprostanoligenes_group, Acidaminococcus, Ruminococcaceae_NK4A214_group*
Calcium, fruit	Cyan module	Plasma			*Dialister, f__Ruminococcaceae*
Potatoes, starch, trans fat	Green module	Plasma			*f__Bacteroidales_S24-7_group*
Fiber, vegetables, tomatoes	Green module	Stool			*Lachnospira, Lachnoclostridium, f__Prevotellaceae*
Cheese, sodium	Lightcyan module	Plasma			*Parasutterella*
Starch, trans fat	Purple module	Plasma			*f__Bacteroidales_S24-7_group*
Vitamin E	Red module	Plasma			*Prevotella_2*
Tomatoes, vegetables	Red module	Stool			*Christensenellaceae_R-7_group, Lachnospira*
Dairy, sodium, cheese	Salmon module	Plasma			*Paraprevotella, Parasutterella, Phascolarctobacterium*
B carotene, lutein + zeaxanthin, vitamin B12, vitamin E, lysine, sodium, copper	Tan module	Plasma			*Ruminococcaceae_NK4A214_group, Oscillibacter*
Calcium, lactose, dairy, vitamin D, vitamin B12	Turquoise module	Plasma			*Dialister*
Fiber	Turquoise module	Stool			*[Eubacterium]_coprostanoligenes_group*

*^∗^Bacterium reported as genus, unless otherwise specified (f, family; c, class; o, order; p, phylum; k, kingdom).*

## Discussion

The gut microbiome is recognized as a key intermediate between environmental inputs and host metabolism, however, the specific relationship between dietary nutrients, microbiome composition, and host metabolism remains poorly understood. We conducted multi-omic profiling to probe the relationship between diet, the microbiome, and metabolism in healthy adults. We identified associations between diet, the gut microbiome and the gut and plasma metabolome at a global level and identified specific microbiome-mediated associations between diet and metabolites. Our data suggest that gut microbiome composition, both at the taxon and the enterotype level, modulates how dietary nutrients are metabolized, impacting systemic host metabolism with potential downstream consequences on metabolic health.

Diet, the microbiome, and the metabolome are complex, composed of multiple inter-dependent variables, which have independent and combinatorial effects. We first examined these multi-omic datasets on a global level, to understand the inter-relationships on a broad scale. Consistent with our hypothesis, diet, the gut microbiome, and the metabolome were all related to each other. We found minimal evidence of an association between the gut and oral microbiota in the same individuals, which is consistent with previous studies, which have also reported limited overlap between different body sites (Caporaso et al., 2011; Ding and Schloss, 2014). The salivary microbiome in our sample was also not strongly related to diet, or to metabolites. This may reflect both the smaller sample size for the oral microbiome, and distal relationships between the mouth and intestinal or whole-body metabolism.

We assessed subjects’ diet using two independent methods, to identify the nutrients consumed shortly before microbiome sampling, and to identify habitual long-term food consumption. There was relatively high correlation between analogous dietary variables from short and long-term estimates within subjects, suggesting that participants’ diets at the time of sampling were consistent with their longer-term dietary patterns. We were interested in the relative importance of day-to-day fluctuations in dietary intake compared with longer-term patterns. We found that long-term diet as assessed by FFQ was more strongly associated with the gut microbiome than the diet consumed immediately prior to sampling (generally the 3 days prior to stool elimination). This suggests a core gut microbial population, shaped by habitual diet, that remains relatively constant despite short-term dietary fluctuations. This is supported by findings from others, who have observed relative stability in gut microbiome profiles over time, particularly in adults (Yatsunenko et al., 2012; Ding and Schloss, 2014; Dubois et al., 2017; Ruggles et al., 2018). Although large shifts in diet acutely alter microbiome composition (David et al., 2014), dietary habits over time appear to be more influential in shaping the gut microbial community. Short-term diet was more strongly associated with the gut and plasma metabolome than long-term diet, independent of the microbiome. This is consistent with a model where recently-consumed nutrients are rapidly metabolized by the host, influencing what is present in the gut and circulation at any given time. However, whether these short-term dynamic changes impact longer-term health outcomes is unknown. It is likely that repeated exposures to diet and microbiome derived metabolites over longer time frames have greater impact on lifelong health status.

Of dietary variables associated with microbiome composition and exhibiting microbiome-mediated relationships with metabolites in our sample, a large proportion are derived from plant-based foods. This is consistent with our knowledge of microbiome-mediated digestion. Plants are complex food sources, and contain many diverse nutrients, some of which are already known to interact with the microbiome. Fiber is metabolized by bacteria for production of short-chain fatty acids, which not only provide energy and selective advantages to microbes, but can affect host metabolism and immunity (Furusawa et al., 2013; Vital et al., 2014; Koh et al., 2016; Maier et al., 2017). Individuals consuming diets high in plant-derived fiber have greater microbiome diversity (Schnorr et al., 2014), while diets low in fiber lead to reduced bacterial diversity (Sonnenburg et al., 2016). Many phytochemicals are selectively metabolized by gut microbiota including isoflavones (Rowland et al., 2000; Fernandez-Raudales et al., 2012), while plants are rich sources of many vitamins, including those with known microbial interaction such as Vitamin B3/Niacin (Singh et al., 2014). Symbiotic relationships between the host and the microbiome, and optimal functioning of the holobiont, are dependent on environment, with diet being the archetypal environmental variable (Postler and Ghosh, 2017). In addition to plant foods, which have long been consumed by humans, we observed inter-relationships with artificial sweeteners, which have entered the human diet in relatively recent time. Our data do not resolve whether these have positive or negative consequences on health, but indicate that shifts toward higher consumption of processed foods and lower consumption of complex plant-based foods, common to the Western diet, have potential consequences on the gut microbiota and metabolite production.

We identified many metabolites in plasma and stool that differed by microbiome composition; indeed the majority of metabolites appeared to be influenced by diet, the microbiome, or both. These spanned many biological pathways, but metabolites that were particularly microbiome-sensitive were pathways related to bile acid metabolism, amino acid metabolism, lipid and steroid metabolism, and metabolism of xenobiotics. While direct effects on diet or microbe-derived metabolites (e.g., xenobiotics) are to be expected, our data highlight that the microbiome also modulates key host metabolic pathways of importance not only for energy metabolism, but overall host health status including immune function. The consequences of alterations in these circulating metabolites are not fully known. Microbiome metabolites have been shown to affect inflammation and immune regulation (Levy et al., 2017; Haase et al., 2018), and we observed some association between enterotype-mediated metabolism and plasma CRP. However, further studies are needed to establish consequences of chronic alterations in metabolite signaling.

Because different bacteria can have overlapping functionality, it can be helpful to collapse the taxonomic composition into related clusters, or enterotypes, to identify individuals within sub-groups of similar composition. We observed many enterotype-mediated associations, amongst them, a significant effect of gut enterotype on the relationship between dietary fiber and plasma bile acids. Bile acids are key regulators of hepatic and intestinal lipid metabolism, and have been linked to inflammation and metabolic disease (Joyce and Gahan, 2016; Chávez-Talavera et al., 2017). Microbiota contribute to bile acid metabolism, transforming host-synthesized primary bile acids to secondary bile acids, while microbiome composition may itself be shaped by bile acids (Wahlström et al., 2016; Long et al., 2017). While we present data for dietary fiber, very similar results were found for dietary choline, with fiber and choline intake strong correlated in our sample. Thus, it is not clear whether the effect is specific to fiber, choline, or another phytonutrient common to the same food source. Both fiber and choline can act as substrates or inhibitors of bile acid metabolism (Corbin and Zeisel, 2012; Dziedzic et al., 2015; Wang et al., 2017), and both have been linked to microbial metabolism (LeBlanc et al., 1998; Tang et al., 2013; Mayengbam et al., 2018; Tuncil et al., 2018), suggesting that either or both plausibly lie in a causal pathway linking diet to bile acid metabolism through microbiota.

Our study had several key strengths, but also some limitations. We recruited healthy adults, and conducted deep multi-omic phenotyping, with the goal of identifying relationships between diet, the microbiome and the metabolome independent of a disease background. While this allowed for metabolic analysis independent of disease confounding or reverse causation, it did not allow us to directly assess relationships with cardiometabolic disease. However, at least half of the participants in our study are likely to develop cardiometabolic disease in later life (Benjamin et al., 2018), suggesting that even mildly elevated risk factors may predict future disease. We measured plasma CRP, as a clinically-relevant marker of inflammation, which predicts future disease risk (Ridker, 2003), and used BMI as a proxy for obesity and future metabolic risk (Van Gaal et al., 2006). Despite our modest sample size, this is one of the largest studies of diet, the microbiome, and the metabolome conducted in humans. A pervasive limitation in nutritional studies is the difficulty in precise quantification of dietary intake in free-living humans. We used two independent validated dietary assessment methods, which were broadly consistent with each other, while allowing us to assess diet over different time frames. Because food is complex, and individual nutrients often co-occur in the same foods, in many cases we can not determine which food component is “causal” in a diet-microbiome-metabolite relationship. Future detailed studies to isolate individual nutrients will be required, while recognizing that nutrients exist within a complex food structure, and that an isolated nutrient (e.g., in a single supplement) may not behave the same way as a nutrient derived in conjunction with other nutrients in a food source. An important limitation of our study is the use of a single time point for data collection. While we were able to identify diet-microbiome-metabolite associations in our cross-sectional analysis, we are unable to infer causality. Future interventional studies with longitudinal sampling are required to assess relationships over time, and to determine whether changes in diet associate with microbiome-mediated changes in metabolism.

## Conclusion

Through multi-omic analysis in a deeply-phenotyped human sample, we identified microbiome-mediated relationships between diet and circulating metabolites. Both individual microbial taxa, and microbial enterotype may relate to how dietary precursors are metabolized within the gut, and in the circulation. The potential mechanisms involved, and any long-term consequences on health status remain to be determined.

## Ethics Statement

This study was carried out in accordance with the recommendations of the University of Pennsylvania’s clinical research standards that meet regulations relating to Good Clinical Practice (GCP). All subjects gave written informed consent in accordance with the Declaration of Helsinki. The protocol was approved by the Institutional Review Boards of the University of Pennsylvania and Vanderbilt University.

## Author Contributions

JF designed the study. HS and JF performed laboratory analysis. Z-ZT, GC, QH, SH, MS, and JF performed statistical analysis. Z-ZT, GC, RS, and JF contributed to writing the manuscript. All authors contributed to manuscript revision, read and approved the submitted version.

## Conflict of Interest Statement

The authors declare that the research was conducted in the absence of any commercial or financial relationships that could be construed as a potential conflict of interest. The reviewer AA declared a past co-authorship with several of the authors Z-ZT and GC.
